# The evolution of sepsis care: from protocol-driven management to personalized intensive care

**DOI:** 10.1007/s15010-026-02852-5

**Published:** 2026-06-01

**Authors:** Tim Rahmel, Benedikt H. Siegler, Markus A. Weigand, Patrick Meybohm, Sascha David, Frank Brunkhorst, Mathias Pletz, Michael Adamzik

**Affiliations:** 1 Deutsche Sepsis-Gesellschaft, Berlin, Germany; 2 Klinik für Anästhesiologie, Intensivmedizin und Schmerztherapie, Ruhr-Universität Bochum, Knappschaft Kliniken Universitätsklinikum Bochum, Bochum, Germany; 3 Medical Faculty Heidelberg, Department of Anesthesiology, Heidelberg University, Heidelberg, Germany; 4 Klinik und Poliklinik für Anästhesiologie, Intensivmedizin, Notfallmedizin und Schmerztherapie, Universitätsklinikum Würzburg, Würzburg, Germany; 5 Institut für Intensivmedizin des Universitätsspital Zürich, Zürich, Switzerland; 6 Institut für Infektionsmedizin und Krankenhaushygiene, Universitätsklinikum Jena, Jena, Germany; 7 Ruhr-Universität Bochum Klinik für Anästhesiologie, Intensivmedizin und Schmerztherapie Knappschaft Kliniken - Universitätsklinikum Bochum, In der Schornau 23-25, 44892 Bochum, Germany

**Keywords:** Sepsis, Precision Medicine, Clinical Practice Guidelines, Personalized Intensive Care Medicine, Refractory Septic Shock

## Abstract

****Purpose**:**

Sepsis remains one of the leading causes of morbidity and mortality worldwide. Despite evolving supportive therapeutic strategies, outcomes, particularly in severe disease manifestations, remain unsatisfactory. In recent years, the conceptual understanding of sepsis has undergone a fundamental shift: from a uniform disease entity to a heterogeneous syndrome characterized by variable pathophysiology and diverse clinical trajectories. In parallel, key aspects of diagnosis and management have been re-evaluated in the context of recently updated guidelines as well as international consensus statements.

****Methods**:**

This review integrates recent guideline updates and emerging concepts in sepsis management into a clinically oriented synthesis. Particular focus is placed on evolving approaches to infection control, hemodynamic stabilization, tissue perfusion–oriented resuscitation, individualized antimicrobial strategies, and the concept of refractory septic shock.

****Results**:**

In the early phase, sepsis management continues to rely predominantly on standardized interventions focused on rapid infection control and hemodynamic stabilization. As the disease progresses, however, therapeutic strategies increasingly require continuous reassessment and adaptation to the individual patient context. Recent guideline developments therefore reflect a broader shift from rigid protocol-driven algorithms toward more flexible, context-sensitive, and physiology-guided approaches. A central component of this evolution is the growing emphasis on tissue perfusion–oriented resuscitation, individualized hemodynamic management, and risk-adapted antimicrobial strategies. Another important recent development is the proposed definition of refractory septic shock, which aims to identify a subgroup of patients with persistent hemodynamic instability and tissue hypoperfusion despite guideline-based initial therapy. Beyond its prognostic relevance, this concept may provide a pragmatic framework for structured therapeutic escalation and phenotype-guided treatment strategies.

****Conclusion**:**

Overall, contemporary sepsis care is evolving toward a staged management model in which standardized early interventions are increasingly complemented by individualized and dynamically adapted therapeutic decisions during later disease stages. Building on these developments, this review integrates recent guideline updates and emerging concepts into a clinically oriented synthesis and discusses their implications for personalized and phenotype-guided sepsis care.

**Supplementary Information:**

The online version contains supplementary material available at https://doi.org/10.1007/s15010-026-02852-5.

## Introduction

 Sepsis remains one of the leading causes of morbidity and mortality worldwide, affecting millions of patients each year [1]. Despite decades of research and continuous refinement of supportive therapeutic strategies, outcomes remain unsatisfactory, particularly in septic shock [2, 3]. Traditionally, the management of sepsis has been shaped by standardized protocols and so-called “bundles”, emphasizing early diagnosis and prompt initiation of therapy [4]. While these uniform strategies have undoubtedly improved initial care, they reach their limitations beyond the acute phase, particularly considering the marked heterogeneity of the sepsis syndrome.

In 2025 and 2026, a notable consolidation of new evidence has emerged. With the update in 2025 of the German S3 guideline by the Deutsche Sepsis-Gesellschaft (DSG 2025) [5] and the release of the new international Surviving Sepsis Campaign guideline in 2026 (SSC 2026) [6], key aspects of sepsis diagnosis and management have been re-evaluated. Classical guideline recommendations are increasingly being complemented by concepts that place greater emphasis on patient-specific factors, perfusion-based parameters, and dynamic therapeutic decision-making. In parallel, the international consensus statement on the definition of refractory septic shock represents a decisive step toward the phenotypic stratification of severe sepsis courses [7]. Taken together, these developments reflect a shift from predominantly protocol-based management toward more adaptive and context-sensitive therapeutic strategies.

The aim of this review is to systematically contextualize these recent developments, to delineate similarities and differences between national and international guidelines, and to analyze the clinical relevance of the concept of refractory septic shock within the broader transition toward individualized and phenotype-guided sepsis care. Furthermore, this article proposes an integrative conceptual framework that illustrates the evolution of sepsis management from predominantly protocol-driven strategies toward increasingly personalized intensive care medicine.

## Evolution of clinical guidelines—from rigid protocols to individualized therapy

Modern sepsis management is historically closely linked to the development of standardized treatment protocols. In particular, the SSC has played a pivotal role over the past two decades in establishing structured and time-sensitive care for patients with sepsis. Previous sepsis guidelines focused on organizing management along clearly defined care bundles, thereby enabling standardization of initial therapy [8, 9]. This approach primarily aimed to reduce delays in treatment initiation and to harmonize the quality of care across different healthcare settings. However, with increasing clinical experience and a growing body of evidence, it has become clear that sepsis is not a uniform disease entity but rather a highly heterogeneous syndrome characterized by variable pathophysiology, diverse sources of infection, and markedly divergent clinical trajectories [10–14]. The strength of standardized protocols, namely their clarity and simplicity, has simultaneously proven to be a limitation, as they insufficiently account for individual variability. In the context of complex and dynamic sepsis states, it is increasingly recognized that strictly protocol-based management does not invariably lead to optimal outcomes [15].

Against this background, DSG 2025 represents an important step, further refining existing concepts and aligning them more closely with the clinical reality of sepsis as a heterogeneous syndrome [5]. Methodologically, it remains committed to the GRADE system and builds upon international evidence, while incorporating national particularities and practice-oriented considerations. Notably, there is a stronger emphasis on clinical decision-making processes rather than solely rigid recommendations. For example, novel diagnostic and therapeutic approaches are integrated that enable earlier and more flexible intervention. For example, the recommendation to initiate vasopressor therapy via peripheral access exemplifies this shift, offering a pragmatic solution to a common clinical dilemma: delays in hemodynamic stabilization due to awaiting central venous access. In a prospective cohort analysis of the CLOVERS trial, peripheral vasopressor administration was frequently employed and associated with a low complication rate, supporting the safety and feasibility of short-term peripheral vasopressor use in early sepsis management [16]. Similarly, the increased emphasis on parameters such as capillary refill time (CRT) reflects a growing consideration of functional and dynamic aspects of the microcirculation and thus organ perfusion [17–21].

The SSC 2026 builds upon these developments and advances them further [6]. While the methodological foundation remains unchanged, the conceptual focus has shifted substantially. The guideline is no longer intended as a rigid rulebook but rather as a decision-support framework that accounts for varying clinical contexts, resource availability, and patient-specific factors. This underscores that sepsis management is no longer viewed solely through the lens of immediate survival but increasingly incorporates long-term consequences and broader systemic considerations.

Taken together, current guideline developments show a clear shift toward greater contextualization of sepsis care. Whereas earlier recommendations primarily focused on standardization and process optimization, contemporary approaches increasingly emphasize adaptation to the individual clinical context. Guidelines are thus evolving from prescriptive directives into structured frameworks that support clinical decision-making while integrating patient-specific factors. This transformation forms the basis for a progressively individualized approach to sepsis management and paves the way for more advanced concepts of precision medicine.

## Key clinical advances in sepsis management

The changes described in both DSG 2025 and SSC 2026 are directly reflected in clinical practice and are leading to a reorientation of therapeutic principles. These developments are particularly evident along key clinical decision-making pathways, ranging from early diagnosis to hemodynamic stabilization.

Early recognition of sepsis remains an important hallmark, which continues to be critical for clinical outcomes. While earlier approaches emphasized the quick sequential organ failure assessment (qSOFA) [22, 23], there is a growing shift away from the notion that a single tool is sufficient for reliable sepsis screening. As already highlighted in the SSC 2021 guideline, qSOFA should not be used as a stand-alone screening instrument [8]. This trend is further reinforced in the current generation of guidelines, which increasingly emphasize multimodal and continuous screening strategies. In addition, serum lactate has gained particular importance. Lactate is no longer viewed solely as a marker of impaired tissue oxygenation or as a prognostic indicator. Instead, lactate clearance is increasingly recognized as early indicator of successful hemodynamic resuscitation. This allows for a more individualized assessment of the therapeutic response.

In parallel, hemodynamic management has undergone substantial evolution. Whereas earlier paradigms were largely characterized by standardized fluid resuscitation strategies, contemporary approaches increasingly favor flexible, context-adapted hemodynamic assessment followed by individualized circulatory support. One of the most important advances is the early initiation of vasopressor therapy, including administration via peripheral venous access. This approach enables rapid hemodynamic stabilization without delaying treatment initiation for central venous catheter placement and reduces the need for excessive fluid administration while awaiting definitive vascular access. Overall, this shift reflects the growing recognition that time is a critical determinant of outcome and that pragmatic, rapidly deployable interventions may offer greater clinical benefit than formally ideal but time-consuming procedures.

Of particular significance is the evolution of therapeutic targets from rigid macrocirculatory parameters to markers of tissue perfusion. Traditionally, hemodynamic resuscitation has been guided primarily by variables such as mean arterial pressure (MAP). However, it is now well established that normalization of pressure does not necessarily equate to adequate microcirculatory perfusion [24]. The incorporation of CRT as a bedside clinical parameter underscores this paradigm shift [17, 24]. It provides a simple and readily available assessment of peripheral perfusion and complements established markers such as lactate. However, important methodological limitations of CRT assessment must be acknowledged underscoring the complexity of this apparently simple bedside parameter. Most interventional studies have not consistently standardized key variables such as compression time, applied pressure, or measurement site, despite evidence that these factors may substantially influence reproducibility and interpretation [21]. Consequently, broader implementation of CRT-guided strategies will require rigorous methodological standardization to avoid variability and potential misinterpretation. Overall, the DSG 2025 und SSC 2026 recommendations clearly reflects a transition from a pressure-oriented to a perfusion-oriented approach, placing effective tissue oxygen delivery at the center of therapeutic decision-making.

A similar trend toward differentiation is evident in antimicrobial therapy. Early administration of antibiotics to treat the underlying infection remains a cornerstone of sepsis management; however, it is increasingly complemented by strategies that prioritize more precise diagnostics and early de-escalation when appropriate. The SSC 2026 guideline explicitly emphasizes the importance of responsible antibiotic use, thereby integrating principles of antimicrobial stewardship into standard care [25]. The overarching goal is to balance the need for rapid, effective treatment with the avoidance of unnecessary or excessive antibiotic exposure.

In summary, contemporary sepsis management is becoming more differentiated across multiple domains. Early recognition is increasingly continuous, hemodynamic therapy more flexible and pragmatic, and therapeutic success more often assessed using functional parameters of tissue perfusion. These changes reflect a deeper understanding of sepsis pathophysiology and support a more patient-centered approach to therapy.

## Direct comparison of the new sepsis guidelines—between global standardization and national specification

The comparison of recently published sepsis guidelines provides substantial interpretative value. The DSG 2025 is explicitly designed as an update of the former national guideline version of 2018 [26] and adopts the SSC 2021 recommendations [8] as its reference. At the same time, it deliberately extends beyond a mere adaptation by integrating additional German S3 guidelines including those on mechanical ventilation, renal replacement therapy, as well as positioning and mobilization of critically ill patients. Thereby aiming for stronger alignment with national clinical practice. Its methodological framework emphasizes that the DSG 2025 guideline is not intended as a simple translation of international recommendations, but rather as a context-adapted implementation that accounts for country-specific healthcare structures, resources, and clinical practice patterns [5].

With the publication of the SSC 2026 [6], this national adaptation is placed in a new context. The international guideline not only updates individual recommendations but also significantly broadens its scope. It explicitly addresses not only in-hospital management but also prehospital care and the immediate post-discharge phase. In doing so, it more closely links acute management with issues of care transitions and the substantial long-term sequelae of sepsis [3]. As outlined in its introduction, the SSC 2026 guideline aims to support the management of adult patients with sepsis across the entire continuum of care, while facilitating both patient-centered decision-making and improvements in healthcare systems [6].

The differences between DSG 2025 and SSC 2026 can largely be attributed to three factors: (i) temporal differences in the underlying evidence base, (ii) the differing scope of an international versus a national guideline, and (iii) varying degrees of willingness to translate limited or emerging evidence into concrete clinical recommendations (Table 1). The latter aspect is particularly characteristic of DSG 2025. Whereas the SSC 2026 framework adopts a comparatively cautious, broadly applicable, and system-oriented approach, DSG 2025 tends to provide more explicit, operational, and practice-oriented recommendations aimed at facilitating bedside implementation. Key differences between DSG 2025 and SSC 2026 are summarized in Table 1, while a detailed side-by-side comparison of selected original recommendation statements, including recommendation strength and evidence grading, is provided in Supplementary Table S1.

**Table 1:** Comparison of Key Aspects of Sepsis Guidelines (SSC 2021 vs. DSG 2025 vs. SSC 2026)

Domains	SSC 2021	DSG 2025	SSC 2026
Guideline Characteristics	Global, evidence-based, focused on the acute phase	National S3 guideline, adapted from SSC 2021, with integration of German specialty guidelines	Globally expanded, system-oriented (including prehospital care, post-acute phase, and recovery)
Screening and Early Recognition	Performance improvement programs recommended; qSOFA not endorsed as a sole screening tool	Recommendation of structured screening programs; no fixed tool, pragmatic selection	Continuation of multimodal strategies; stronger integration into care pathways
Antimicrobial Therapy	Immediate antibiotic therapy in septic shock; 3 h approach in suspected sepsis without shock	“As early as possible” in sepsis; within 1 h in septic shock; up to 3 h in suspected sepsis without shock	Refined risk stratification: within 1 h for high probability (even without shock), within 3 h for possible sepsis, and a more cautious approach in low-probability cases
Antibiotic Spectrum	Initial broad-spectrum coverage	More restrictive; no routine Gram-negative combination therapy	Risk-adapted; Gram-negative combination therapy may be considered in high-risk patients
Antifungal Therapy	Selective use in high-risk patients	Clearly binary approach: risk present vs. absent; antifungal therapy accordingly	Differentiated strategy based on risk profile and biomarkers
Antimicrobial Stewardship	Implicitly addressed	Partially integrated, but less system-oriented	More strongly integrated; explicit focus on balancing early therapy with avoidance of overtreatment
Fluid Therapy	Crystalloids, initial 30 mL/kg (with limitations)	Crystalloids with individualized application	Crystalloids, with greater individualization and context-specific use
Type of Fluids	Crystalloids preferred	Crystalloids; no gelatin; albumin when indicated	Crystalloids; no specification of fluid subtype, no albumin
Hemodynamics – Target Values	MAP = 65 mmHg	MAP ≥ 65 mmHg	MAP ≥ 65 mmHg, with individualization (e.g., 60–65 mmHg in patients > 65 y)
Perfusion Parameters	Lactate; capillary refill time mentioned as an adjunct parameter	Lactate plus explicit recommendation of capillary refill time	Greater emphasis on perfusion-guided therapy (lactate, clinical parameters)
Vasopressors	Norepinephrine as first-line; central administration as standard	Norepinephrine as first-line; explicit recommendation for early peripheral administration	Norepinephrine as first-line; more flexible, algorithm-based use
Corticosteroids	Restrictive recommendation in persistent shock	Specific recommendation: hydrocortisone ± fludrocortisone	Suggestion for intravenous corticosteroids; less operationalized
Adjunctive Therapies	Limited negative recommendations	Clear rejection of selected interventions (e.g., vitamin C, adsorption therapies, high-volume hemofiltration)	Broader negative recommendations (vitamin C, IVIG, blood purification, probiotics, etc.)
Mechanical Ventilation and Organ Support	Basic principles, less detailed	Integration of German S3 guidelines → high level of details	Expanded scope, but more principle-based
Post-Acute Care/Recovery	Initial recommendations for discharge and post-acute care	Expanded focus: post-intensive care syndrome (PICS), relatives, discharge management	Substantially expanded: transitions of care, rehabilitation, long-term outcomes, and social aspects
Guideline Philosophy	Focus on standardization and evidence-based care	Practice-oriented, operational, individualization	System-oriented perspective, individualization

*SSC*  Surviving Sepsis Campaign; *DSG * German Sepsis Society; *qSOFA * quick Sequential Organ Failure Assessment; *h *  hour(s); *Gram neg. * Gram-negative; *MAP*  mean arterial pressure; *i.v.* intravenous; *HVHF*  high-volume haemofiltration; *IVIG * intravenous immunoglobulins; *PEEP*  positive end-expiratory pressure; *ECMO *  extracorporeal membrane oxygenation; *PICS * post-intensive care syndrome

This distinction is already evident in screening and early recognition chapters. The SSC 2021 guideline recommended performance improvement programs incorporating sepsis screening, while explicitly rejecting the use of qSOFA as a single screening tool. The DSG 2025 guideline adopts this framework but refines it for the German healthcare context. It recommends that hospitals implement structured quality improvement programs, including locally adapted screening strategies and standard operating procedures for sepsis management. Notably, it refrains from endorsing a single screening tool, thereby acknowledging the limitations of individual scoring systems and emphasizing the need for clinically meaningful, locally implementable solutions. The SSC 2026 guideline reaffirms the importance of effective screening strategies, highlighting NEWS, NEWS2, MEWS, and SIRS as preferred tools, while simultaneously acknowledging that none can be considered optimal. Among these, the NEWS2 score appears to be emerging as the most reliable option in current practice [27, 28].

The comparison between both current guidelines is particularly illustrative in the domain of antimicrobial therapy. Here, the evolution from SSC 2021 to DSG 2025 and further to SSC 2026 is especially apparent. The SSC 2021 guideline already differentiated between septic shock and other clinical scenarios but largely adhered to a two-tiered model balancing immediate treatment in high-risk cases against a more diagnostic-driven approach in uncertain situations. The DSG 2025 guideline largely follows this structure, recommending early intravenous antimicrobial therapy in sepsis and initiation within one hour in septic shock. In cases of suspected sepsis without shock and with an unclear source of infection, further diagnostic evaluation is prioritized, with antibiotic initiation recommended within three hours if suspicion persists. The SSC 2026 guideline advances this concept not through uniform acceleration of treatment, but by introducing a more refined clinical risk stratification. It more explicitly differentiates according to infection probability and severity, thereby acknowledging the tension between lifesaving early antibiotic therapy and the risks associated with unnecessary antibiotic use. In this framework, initiation within one hour is recommended for moderate to high infection probability, whereas a more cautious approach with extended diagnostic evaluation is reserved for low-probability cases. In contrast, DSG 2025, similar to SSC 2021, remains more conservative in non-shock sepsis, allowing up to three hours and thereby placing greater emphasis on diagnostic clarification.

Beyond timing, both guidelines increasingly emphasize optimization of antimicrobial exposure. The SSC 2026 guideline strongly recommends prolonged or continuous beta-lactam infusion following an initial loading dose, supported by recent randomized evidence, a strategy that had already been anticipated in DSG 2025 [29]. In parallel, therapeutic drug monitoring (TDM) is further integrated into clinical decision-making. While SSC 2026 recommends TDM on a case-by-case basis, DSG 2025 places greater emphasis on its use in critically ill patients with substantial pharmacokinetic variability, particularly during prolonged or continuous beta-lactam infusion. This approach is supported by growing evidence demonstrating frequent failure to achieve pharmacokinetic/pharmacodynamic target attainment with standard dosing regimens in critically ill patients, while TDM-guided strategies may improve individualized dosing and reduce potentially excessive antibiotic exposure, although robust evidence for patient-centered outcome benefits remains limited [30–32]. At the same time, antimicrobial stewardship is more explicitly embedded into therapeutic recommendations. In addition, it introduces a conditional recommendation for selective digestive decontamination in mechanically ventilated patients in settings with low prevalence of multidrug-resistant organisms, a strategy not addressed in DSG 2025 [5]. Regarding treatment duration, SSC 2026 conditionally supports procalcitonin-guided shortening of antibiotic therapy while advising against its use for treatment initiation, whereas DSG 2025 issues a stronger recommendation for PCT-guided duration. Taken together, these developments reflect a shift from a primarily empiric, broad-spectrum approach toward a more differentiated, risk-adapted, and ecology-aware antimicrobial strategy.

In hemodynamic management, similarities between the guidelines initially predominate. Both recommend balanced crystalloids as first-line therapy, favor balanced solutions, advocate dynamic over static assessments of fluid responsiveness, and identify norepinephrine as the vasopressor of choice. However, differences in emphasis are evident. The DSG 2025 guideline is more operational and practice-oriented, for example recommending early peripheral vasopressor administration to rapidly restore MAP without delaying treatment for central venous access. It also highlights CRT as a clinically relevant parameter for assessing tissue perfusion in septic shock. The SSC 2026 guideline continues to recommend an initial MAP target of approximately 65 mmHg in septic shock but introduces age-adapted modifications, suggesting a lower target range of 60–65 mmHg in patients aged 65 years or older. This reflects a more individualized approach to hemodynamic management within a defined target range. In contrast, the DSG 2025 guideline provides more direct and operational bedside guidance by emphasizing fixed hemodynamic thresholds and pragmatic implementation strategies. Rather than being fully interchangeable approaches, these recommendations reflect different conceptual priorities. The SSC 2026 framework places stronger emphasis on individualized target adaptation, whereas DSG 2025 prioritizes practical standardization and bedside applicability. Notably, both approaches ultimately pursue the same overarching goal of adequate tissue perfusion and individualized patient stabilization.

A similar pattern is observed in corticosteroid therapy. The SSC 2026 guideline maintains a cautious recommendation for intravenous corticosteroids in septic shock with persistent vasopressor requirements. The DSG 2025 guideline translates this into more concrete clinical practice, explicitly recommending hydrocortisone with or without fludrocortisone and defining the clinical context more precisely. This exemplifies the strength and limitation of the DSG 2025 guideline: it enhances clinical applicability while potentially underrepresenting the increasing need for individualized treatment strategies.

The distinct profile of DSG 2025 is even more evident in its approach to adjunctive therapies. The DSG 2025 guideline adopts clear positions on several commonly used but weakly supported interventions. It recommends against the use of high-dose vitamin C, both as monotherapy and in combination, advises against unselected adsorption therapies, and rejects high-volume hemofiltration. For other interventions, it refrains from making definitive recommendations due to insufficient evidence and still ongoing RCTs in a given research field. The SSC 2026 guideline adopts an even broader perspective, recommending against a range of additional interventions, including intravenous vitamin C, immunoglobulins, various blood purification techniques, vitamin D, and probiotics. This clarity is particularly valuable in clinical settings where technical feasibility may be mistaken for clinical efficacy. Both guidelines converge on a central message: routine use of adjunctive therapies is currently not justified. Only with improved translation of precision medicine concepts into clinical practice will it become possible to tailor such therapies to appropriate patient subgroups.

In the domains of mechanical ventilation, organ support, and post-sepsis care, the differences between DSG 2025 and SSC 2026 are best understood as expressions of distinct guiding philosophies. The DSG 2025 guideline gains depth through the integration of national specialty guidelines, enabling more detailed and context-specific recommendations. In contrast, the SSC 2026 gains breadth by conceptualizing sepsis care as a continuum, placing greater emphasis on transitions of care, rehabilitation, and long-term outcomes. While DSG 2025 has already expanded its post-sepsis care section compared with earlier versions, the SSC guideline advances it further by framing sepsis management as a comprehensive care pathway from prehospital recognition through acute treatment to post-discharge recovery. This domain likely represents the clearest indicator of future guideline development.

A distinctive feature of the DSG 2025 guideline is its dedicated chapter on infection prevention and vaccination, which has no equivalent in SSC 2026 and underscores its independent clinical profile. DSG 2025 recommends multimodal prevention strategies, including catheter bundles, hand hygiene, and structured antimicrobial stewardship. Importantly, it explicitly integrates vaccination—according to STIKO recommendations—as a core sepsis prevention strategy, targeting influenza, COVID-19, pneumococci, meningococci, and Haemophilus influenzae type B. Annual vaccination of healthcare workers is advised to reduce nosocomial transmission, while high-risk populations, such as asplenic patients, receive tailored preventive measures including emergency documentation and standby antibiotics. In contrast, SSC 2026 focuses exclusively on the management of established sepsis and does not address prevention. The inclusion of vaccination in DSG 2025 reflects a broader conceptualization of sepsis as a preventable condition and aligns with growing evidence that immunization reduces both sepsis incidence and downstream complications. From a precision medicine perspective, this approach represents a shift toward risk-adapted prevention, targeting defined vulnerable populations and pathogen-specific threats.

For clinical practice, these two guidelines should be viewed as complementary. The DSG 2025 remains the central and highly relevant framework for clinical decision-making within Germany, while the SSC 2026 highlights the direction of international development toward a more individualized, continuum-based model of care. Consequently, future national updates will likely need to extend beyond revising individual recommendations to systematically translating post-acute and longitudinal care concepts into the national healthcare context. Taken together, these developments indicate that early sepsis management remains grounded in standardized, protocol-based care, whereas later phases increasingly require context-adapted decisions. Both DSG 2025 and SSC 2026 exemplify that the increasing recognition of disease heterogeneity is driving a move toward more individualized and context-adapted strategies, thereby providing the foundation for explicit phenotypic stratification.

## Refractory septic shock—from a poorly defined clinical problem to an action-guiding entity

A key clinical manifestation of this heterogeneity becomes particularly evident in septic shock as the most severe manifestation of sepsis and is associated with particularly high mortality [33]. However, substantial heterogeneity exists within this patient population. A subset of patients fails to respond adequately to guideline-based initial therapy and develops persistent hemodynamic instability. In clinical practice, the term “refractory septic shock” is used to describe this condition, although a uniform definition has been lacking. This ambiguity has not only complicated clinical communication but has also hindered the design and interpretation of clinical studies.

With the recent SCCM/ESICM consensus statement, a structured attempt has been made for the first time to define refractory septic shock using pragmatic clinical criteria [7]. Importantly, this concept is currently not incorporated into either the DSG 2025 or SSC 2026 guidelines and should primarily be understood as a clinically oriented conceptual framework intended to support research and bedside stratification rather than a definitive diagnostic entity. While the consensus identified key clinical characteristics of refractory septic shock, no agreement was reached regarding the exact duration of shock persistence or the extent and duration of prior therapeutic interventions required before classification. Consequently, refractory septic shock should presently be regarded as a pragmatic construct identifying patients who fail to adequately respond to initial guideline-based therapy and who therefore require structured reassessment and individualized therapeutic escalation.

At the same time, the concept reflects the increasing recognition of sepsis heterogeneity and may support the identification of a clinically meaningful high-risk phenotype characterized by persistent tissue hypoperfusion despite adequate initial resuscitation. Central elements include failure to normalize elevated lactate levels and/or prolonged capillary refill time despite restoration of target MAP, lack of fluid responsiveness, and high vasopressor requirements, typically exceeding norepinephrine equivalents of 0.5 µg/kg/min [7]. Whether refractory septic shock represents a heterogeneous collection of biologically distinct phenotypes shaped by host factors or rather a common terminal pathway of dysregulated immunologic and hemodynamic responses remains unresolved and will require further investigation.

**Fig. 1:**
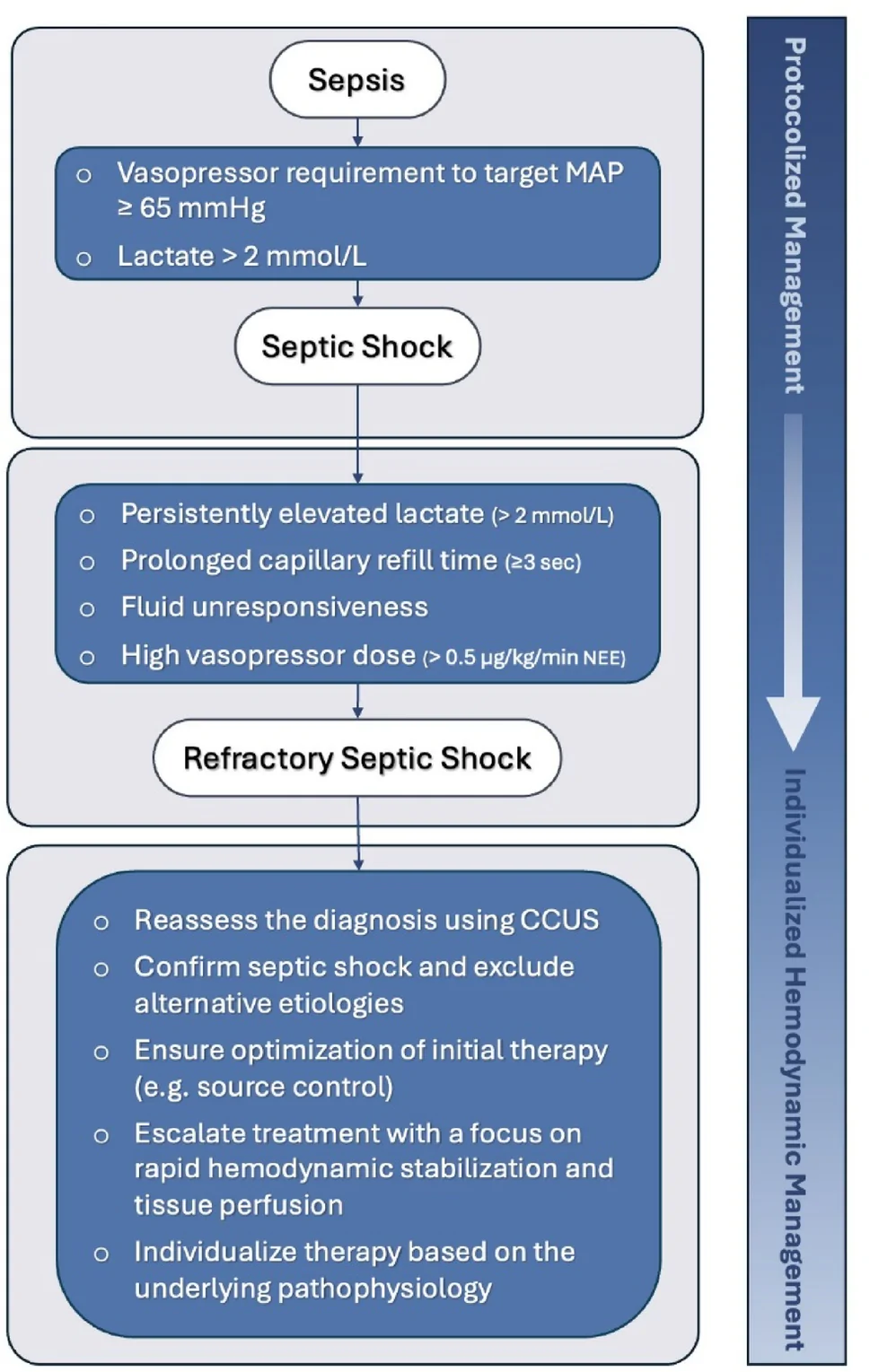
Clinical framework for the identification and management of refractory septic shock. The figure illustrates the progression from sepsis to septic shock according to established definitions (Sepsis-3, SSC 2021/2026, DSG 2025) and highlights the transition toward refractory septic shock as defined by the recent SCCM/ESICM consensus. Septic shock is characterized by the need for vasopressor therapy to maintain a mean arterial pressure (MAP) ≥ 65 mmHg in combination with elevated serum lactate levels (> 2 mmol/L). Refractory septic shock represents a distinct high-risk subgroup defined by persistent tissue hypoperfusion despite adequate initial resuscitation, reflected by persistently elevated lactate levels and/or prolonged capillary refill time, lack of fluid responsiveness, and high vasopressor requirements (typically norepinephrine equivalents > 0.5 µg/kg/min). *MAP*  mean arterial pressure; *CCUS*  critical care ultrasound; *NEE* norepinephrine equivalent.

For clinical practice, it is essential that this definition is not regarded merely as a descriptive construct, but as a decision-making tool to guide therapeutic escalation beyond standard care. Refractory septic shock thus identifies a subgroup of patients in whom escalation, e.g. by targeted adjunctive treatments, should be actively considered. This reframes refractory septic shock as an action-guiding clinical entity that requires structured reassessment and therapeutic escalation. From a pragmatic standpoint, the presence of these criteria necessitates a structured reassessment of the clinical situation *(*Fig. 1*)*.

An important aspect of refractory septic shock is that its assessment extends beyond isolated macrocirculatory parameters such as MAP or vasopressor dose and incorporates markers of tissue perfusion, including lactate kinetics and capillary refill time. This reflects the broader shift toward perfusion-oriented resuscitation strategies discussed above. From a practical perspective, persistent abnormalities in these parameters despite apparent hemodynamic stabilization should prompt structured reassessment and consideration of therapeutic escalation. While standardized interventions dominate the early phase of sepsis management, refractory septic shock requires a more individualized approach. This may include the early combination of different vasopressors, careful fine-tuning of fluid administration, the use of inotropic agents, or the targeted application of adjunctive therapies. In selected cases, advanced modalities such as extracorporeal support systems and adjunctive therapies may be considered. Importantly, the emphasis is less on any single intervention than on aligning therapeutic strategies with the patient’s underlying pathophysiology and endotype.

Finally, the proposed criteria provide an opportunity to identify a clearly defined high-risk subgroup suitable for targeted clinical research. Beyond their research implications, they may also support bedside recognition of patients who are unlikely to respond adequately to standard initial therapy. In this sense, refractory septic shock can be understood as a first pragmatic step toward phenotype-based stratification in sepsis care.

## Integration—toward phenotype-guided sepsis therapy

Building on this development, the updated clinical guidelines [5, 6] and the recent definition of refractory septic shock [7] should not be viewed as independent advances, but as complementary elements of a broader change in sepsis care. Current guidelines increasingly move away from rigid algorithms and instead provide flexible decision support for different clinical contexts. Refractory septic shock illustrates how this approach can be applied at the bedside by identifying patients in whom standard initial therapy is insufficient and further escalation should be considered. This perspective is supported by recent trials in which appropriate patient selection appears to be a key determinant of therapeutic success [34–36]. The ImmunoSep trial represents one of the first large-scale efforts to operationalize precision medicine concepts in sepsis through biomarker-guided immunomodulatory therapy [34]. In this multicenter randomized trial, patients were stratified according to predefined immune profiles with the aim of matching immunologic endotypes to targeted interventions, thereby demonstrating the feasibility of individualized immune-based therapeutic strategies in sepsis. Similarly, the ANDROMEDA-SHOCK-2 trial evaluated a personalized hemodynamic resuscitation strategy targeting capillary refill time as a physiologically informed bedside marker of tissue perfusion [35]. Compared with more conventional resuscitation approaches, this strategy emphasized dynamic and individualized adjustment of circulatory support based on peripheral perfusion assessment rather than relying predominantly on macrocirculatory variables alone. In parallel, the TIGRIS trial investigated polymyxin B hemoadsorption in patients with endotoxic septic shock using a more biologically enriched patient selection strategy [36]. Beyond the evaluation of a specific adjunctive therapy, the study further highlights the growing importance to identify distinct sepsis subgroups potentially amenable to targeted therapeutic interventions.

Sepsis management is increasingly shaped by biologically informed patient stratification and dynamically adapted therapeutic strategies. Rather than applying uniform treatment algorithms across all patients, contemporary approaches seek to account for differences in host response, hemodynamic profiles, and disease trajectories. In this context, refractory septic shock may represent a clinically important high-risk subgroup in whom standard initial interventions are insufficient and escalation toward adjunctive or rescue therapies should be considered [11, 37].

**Fig. 2:**
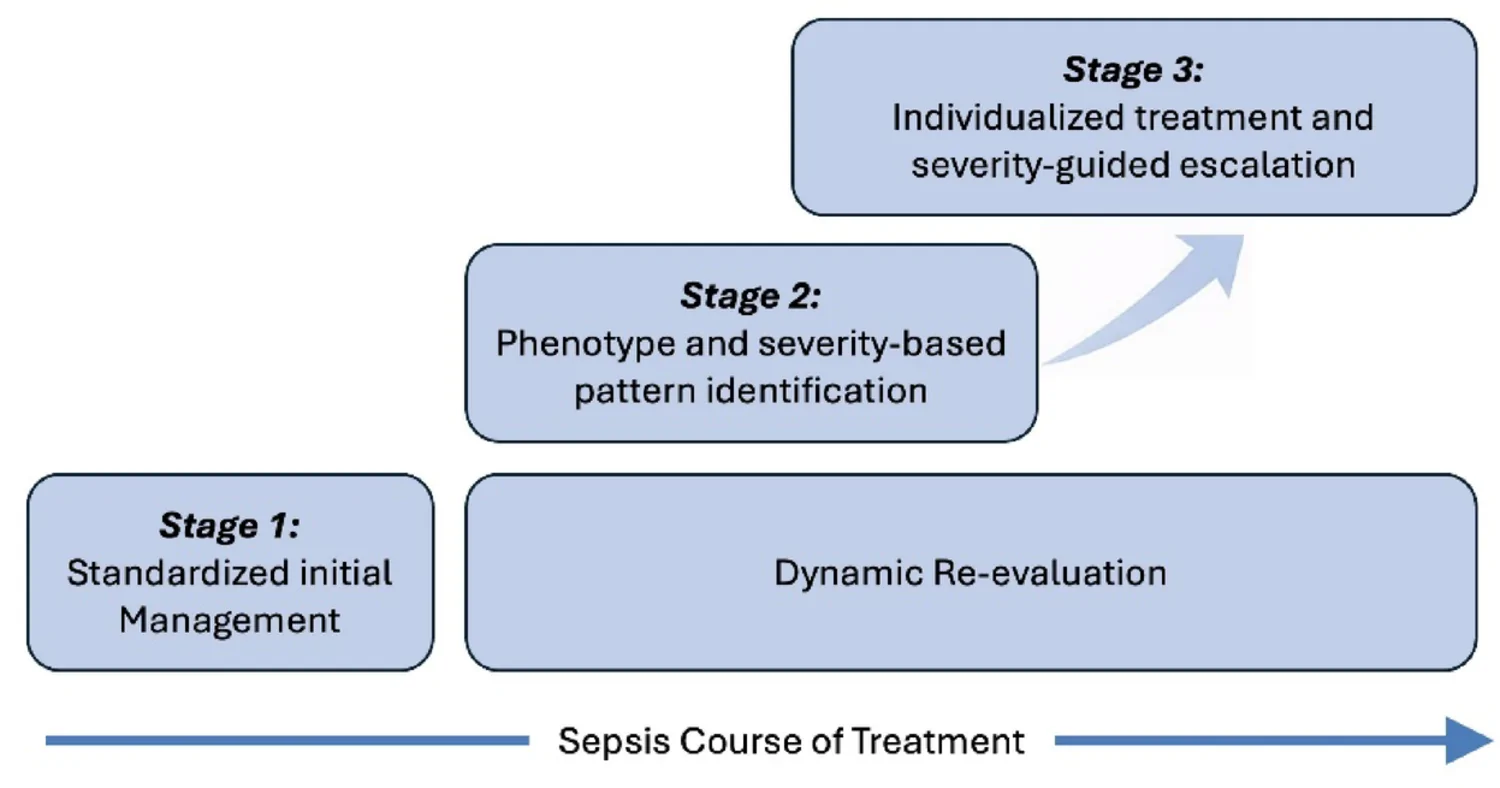
Conceptual model of the transition from standardized to phenotype- and severity-guided sepsis therapy. Staged model of sepsis management illustrating the transition from standardized early care to phenotype-guided individualized therapy. Continuous re-evaluation links initial protocol-based treatment with later, patient-specific therapeutic adaptation and escalation

The initial phase of treatment remains characterized by standardized “bundle-based” interventions, as the rapid implementation of evidence-based measures is critical during this stage. The major innovation, however, lies in the subsequent phase, during which treatment requires continuous reassessment and adaptation to the patient’s evolving condition *(*Fig. 2*).* As sepsis progresses and disease complexity increases, individualized therapeutic adjustments become increasingly important. This transition is gradual and guided by indicators of tissue perfusion and organ dysfunction. Within this framework, refractory septic shock may represent the point at which the limitations of standardized therapy and the importance of individual pathophysiology become most apparent.

This conceptual shift has important implications for clinical practice. It requires moving beyond a predominantly uniform treatment paradigm toward more nuanced clinical decision-making in which phenotypic concepts gain increasing relevance. Considerable efforts have therefore been undertaken to identify subgroups of sepsis patients who differ in their pathophysiology and therapeutic responsiveness [11, 34]. In this regard, refractory septic shock may be understood as a clinically meaningful phenotype characterized by profound hemodynamic dysregulation and persistent microcirculatory dysfunction [7]. Unlike complex molecular classification systems, this concept is immediately applicable at the bedside and therefore directly translatable into clinical practice. Accordingly, the ability to interpret dynamic clinical trajectories becomes increasingly important. In summary, refractory septic shock may represent the clearest clinical manifestation of the transition from protocolized sepsis care toward highly individualized precision-based management.

## Conclusion—from standardized care to individualized sepsis therapy

Modern sepsis management is undergoing a transition from a rigid predominantly protocol-based approach toward a dynamic and adaptive model of sepsis care. Structured initial management remains fundamental, comprising early diagnosis, prompt antimicrobial therapy, and hemodynamic stabilization. However, the key transformation concerns the phase following initial stabilization. It is at this stage that the subsequent clinical course is determined and where the need for individualized therapy becomes most evident. Accordingly, sepsis management must be understood as a continuous process requiring ongoing re-evaluation.

In clinical practice, this shift is reflected in a transition from a purely pressure-oriented approach to one guided by perfusion and organ function. Parameters such as lactate kinetics, capillary refill time, and organ function are increasingly prioritized over isolated pressure target values. The concept of refractory septic shock provides an important clinical anchor in this context. It identifies patients in whom standard interventions are insufficient and marks the transition to a structured diagnostic and therapeutic escalation. At the same time, antimicrobial therapy requires a more risk-adapted and differentiated strategy. Early administration of antibiotics remains critical but must be consistently integrated with principles of antimicrobial stewardship.

Overall, sepsis therapy is evolving into a staged model: standardized during early recognition and initial stabilization, but increasingly adapted during complex disease progression. The practical implication is not a departure from clinical guidelines, but their context-sensitive application. The future of sepsis management thus lies in the systematic integration of clinical dynamics, phenotypic stratification, and precision medicine approaches with the aim of increasingly tailoring therapeutic decisions to the individual patient.

## Supplementary Information

Below is the link to the electronic supplementary material.

Supplementary Material 1

## Data Availability

No datasets were generated or analysed during the current study.
