# Supplementary material for: The evolution of sepsis care: from protocol-driven management to personalized intensive care
Source: Infection. 2026 Jun 1;54(5):2245–55. doi: 10.1007/s15010-026-02852-5 (PMC13727846; doi:10.1007/s15010-026-02852-5)
Supplement: Supplementary file 1 [file 15010_2026_2852_MOESM1_ESM.docx]

# Supplementary Table S1. Comparison of selected recommendation statements from DSG 2025 [1], SSC 2021 [2], and SSC 2026 [3].

This supplementary table summarizes selected recommendations across major domains of sepsis management. Selected recommendation statements were condensed for clarity while preserving conceptual accuracy.

| **Domain** | **Topic** | **DSG 2025** | **Grade** | **SSC 2021** | **Grade** | **SSC 2026** | **Grade** |
| --- | --- | --- | --- | --- | --- | --- | --- |
| Screening | Performance improvement | Quality improvement program including sepsis screening and SOPs. | Strong | Performance improvement program incl. screening and SOPs. | Strong | Performance improvement, screening, SOPs, and quality improvement strategies. | Strong |
| Screening | Screening tools | Use of an appropriate screening tool outside the ICU. | Strong | Against qSOFA alone vs SIRS/NEWS/MEWS. | Strong | Risk-based screening strategies emphasized. | Strong |
| Screening | Lactate | Measure lactate in patients with suspected sepsis. | Strong | Suggest measuring blood lactate. | Weak | Retained as core adjunct. | Conditional |
| Infection | Antibiotic timing | Administer intravenous antimicrobials within 1 hour in septic shock whenever feasible. | Strong | Administer antimicrobials immediately within 1 h. | Strong | Early therapy retained within probability-based framework. | Strong |
| Infection | Possible sepsis without shock | Prompt diagnostics; administer antimicrobials within 3 hours if suspicion persists. | Conditional | Rapid investigation and antimicrobials within 3 h. | Weak | Definite/probable/possible sepsis terminology. | Conditional |
| Infection | Prolonged beta-lactam infusion | Use prolonged or continuous beta-lactam infusion. | Strong | Suggest prolonged beta-lactam infusion. | Weak | Increased PK/PD emphasis. | Conditional |
| Infection | Empiric antifungals | Use empiric antifungal therapy only in high-risk patients. | Weak | Risk-adapted empiric antifungal therapy. | Weak | Recommendation retained. | Conditional |
| Hemodynamics | Balanced crystalloids | Prefer balanced crystalloids. | Weak | Suggest balanced crystalloids. | Weak | Recommendation retained. | Conditional |
| Hemodynamics | Albumin | Use albumin when large fluid volumes are required. | Weak | Suggest albumin after large crystalloid volumes. | Weak | Recommendation retained. | Conditional |
| Hemodynamics | Peripheral vasopressors | Peripheral vasopressors may be initiated before central venous access is established | Conditional | Peripheral vasopressors rather than delaying therapy. | Weak | Recommendation retained. | Conditional |
| Hemodynamics | Vasopressin | Add vasopressin if norepinephrine alone is insufficient. | Weak | Add vasopressin instead of escalating norepinephrine. | Weak | Recommendation retained. | Conditional |
| Hemodynamics | Terlipressin | Do not use terlipressin. | Weak | Suggest against terlipressin. | Weak | Recommendation retained. | Conditional |
| Respiratory | Low tidal volume | Use tidal volumes of approximately 6 mL/kg predicted body weight. | Strong | Low tidal volume ventilation. | Strong | Recommendation retained. | Strong |
| Respiratory | Driving pressure | Target driving pressure ≤14 cmH2O. | Weak | Plateau pressure focused. | Conditional | More individualized ventilation. | Conditional |
| Respiratory | PEEP | Individualize PEEP. | Weak | No explicit individualized recommendation. | No recommendation | More individualized approaches. | Conditional |
| Respiratory | Oxygen targets | Target SpO2 92–96%. | Weak | No recommendation. | No recommendation | Continued uncertainty. | No recommendation |
| Respiratory | Recruitment maneuvers | Do not use prolonged recruitment maneuvers. | Strong | Avoid routine RM. | Weak | Restrictive use retained. | Conditional |
| Respiratory | Prone positioning | Use prone positioning for 12–16 hours. | Strong | Prone positioning in severe ARDS. | Strong | Recommendation retained. | Strong |
| Respiratory | VV-ECMO | Use VV-ECMO only after exhaustion of conservative measures. | Weak | Selected severe ARDS cases. | Conditional | Specialized individualized approach. | Conditional |
| Renal replacement | Timing of RRT | Initiate immediately in the presence of life-threatening derangements. | Conditional | No strong recommendation. | No recommendation | Individualized approach. | Conditional |
| Renal replacement | High-volume hemofiltration | Do not use high-volume hemofiltration. | Weak | Against high-volume hemofiltration. | Weak | Recommendation retained. | Conditional |
| Adjunctive therapies | Corticosteroids | Use hydrocortisone ± fludrocortisone in persistent vasopressor-dependent shock. | Weak | Suggest IV corticosteroids. | Weak | Further strengthened after newer trials. | Conditional |

**Abbreviations:** ARDS = acute respiratory distress syndrome; CRT = capillary refill time; DSG = Deutsche Sepsis-Gesellschaft; ECMO = extracorporeal membrane oxygenation; ICU = intensive care unit; IV = intravenous; IVIG = intravenous immunoglobulins; MAP = mean arterial pressure; MEWS = Modified Early Warning Score; NEWS = National Early Warning Score; PCT = procalcitonin; PEEP = positive end-expiratory pressure; PK/PD = pharmacokinetic/pharmacodynamic; qSOFA = quick Sequential Organ Failure Assessment; RM = recruitment maneuver; RRT = renal replacement therapy; SIRS = systemic inflammatory response syndrome; SOP = standard operating procedure; SpO₂ = peripheral oxygen saturation; SSC = Surviving Sepsis Campaign; TDM = therapeutic drug monitoring; VV-ECMO = veno-venous extracorporeal membrane oxygenation.

## References

1.	Brunkhorst FM, Adamzik M, Axer H, Bauer M, Bode C, Bone HG, et al. [S3 guideline on sepsis-prevention, diagnosis, therapy, and follow-up care-update 2025]. Med Klin Intensivmed Notfmed. 2025;120(2):163-231. <https://doi.org/10.1007/s00063-025-01317-1>.

2.	Evans L, Rhodes A, Alhazzani W, Antonelli M, Coopersmith CM, French C, et al. Surviving sepsis campaign: international guidelines for management of sepsis and septic shock 2021. Intensive Care Med. 2021;47(11):1181-247. <https://doi.org/10.1007/s00134-021-06506-y>.

3.	Prescott HC, Antonelli M, Alhazzani W, Moller MH, Alshamsi F, Azevedo LCP, et al. Surviving Sepsis Campaign: international guidelines for management of sepsis and septic shock 2026. Intensive Care Med. 2026;Online ahead of print. <https://doi.org/10.1007/s00134-026-08361-1>.

1. Brunkhorst FM, Adamzik M, Axer H, Bauer M, Bode C, Bone HG, et al. [S3 guideline on sepsis-prevention, diagnosis, therapy, and follow-up care-update 2025]. Med Klin Intensivmed Notfmed. 2025;120(2):163-231. <https://doi.org/10.1007/s00063-025-01317-1>.

2. Evans L, Rhodes A, Alhazzani W, Antonelli M, Coopersmith CM, French C, et al. Surviving sepsis campaign: international guidelines for management of sepsis and septic shock 2021. Intensive Care Med. 2021;47(11):1181-247. <https://doi.org/10.1007/s00134-021-06506-y>.

3. Prescott HC, Antonelli M, Alhazzanic W, Moller MH, Alshamsi F, Azevedo LCP, et al. Surviving Sepsis Campaign: international guidelines for management of sepsis and septic shock 2026. Intensive Care Med. 2026;Online ahead of print. <https://doi.org/10.1007/s00134-026-08361-1>.
